# Supplementary material for: A vigilant observation to pregnancy associated listeriosis in Africa: Systematic review and meta-analysis
Source: PLOS Glob Public Health. 2022 Oct 14;2(10):e0001023. doi: 10.1371/journal.pgph.0001023 (PMC10021409; doi:10.1371/journal.pgph.0001023)
Supplement: S2 File — (DOCX) [file pgph.0001023.s002.docx]

| Search number | Query | Results | Search Date (17/07/2021) |
| --- | --- | --- | --- |
|  | ("pregnancy"[MeSH Terms] OR "pregnancy"[All Fields]) AND associated[All Fields] AND ("listeriosis"[MeSH Terms] OR "listeriosis"[All Fields]) AND ("Africa"[MeSH Terms] OR "Africa"[All Fields]) | 244 |  |
| 3. | [((pregnancy) AND pregnancy-associated listeriosis) AND Africa](https://www.ncbi.nlm.nih.gov/portal/utils/pageresolver.fcgi?recordid=60fd7f9f1a26692506ee05bb) | 41 |  |

# Exemplary search strategy for PubMed data base
