# Supplementary material for: A vigilant observation to pregnancy associated listeriosis in Africa: Systematic review and meta-analysis
Source: PLOS Glob Public Health. 2022 Oct 14;2(10):e0001023. doi: 10.1371/journal.pgph.0001023 (PMC10021409; doi:10.1371/journal.pgph.0001023)
Supplement: Attachment — Submitted filename: Response letter to editor of PGPH.docx [file pgph.0001023.s003.docx]

Editorial corrections

Dear Editors, we appreciated your efforts and gentle feedbacks and we replied point by points for comments given.

Journal Requirements:

1. Please amend your online Financial Disclosure statement. If you did not receive any funding for this study, please simply state: “The authors received no specific funding for this work.”

**Response:** We have now update the online submission.

2. Please update your online Competing Interests statement. If you have no competing interests to declare, please state: “The authors have declared that no competing interests exist.”

**Response:** We have now updated as directed.

3. Please provide separate figure files in .tif or .eps format and ensure that all files are under our size limit of 10MB.

**Response:** Thanks amendment is done as directed.

4. We noticed that you used “unpublished sources/studies” in the manuscript. We do not allow these references, as the PLOS data access policy requires that all data be either published with the manuscript or made available in a publicly accessible database. Please amend the supplementary material to include the referenced data or remove the references.

**Response**: okay to be clear, it is a searching strategy to mean we have also looked even for unpublished sources/studies at the universities repository. But this does not included unpublished sources to make synthesis, and to be convenient we have updated the eligibility criteria to include only published sources (page4, methods section, and line number 91-92].

5. We have noticed that you have uploaded Supporting Information files, but you have not included a list of legends. Please add a full list of legends for your Supporting Information files after the references list.

**Response:** Thanks for your direction and amendment as required.

6. Please review your reference list to ensure that it is complete and correct. If you have cited papers that have been retracted, please include the rationale for doing so in the manuscript text, or remove these references and replace them with relevant current references. Any changes to the reference list should be mentioned in the rebuttal letter that accompanies your revised manuscript. If you need to cite a retracted article, indicate the article’s retracted status in the References list and also include a citation and full reference for the retraction notice.

**Response:** Thanks for your concern. But we did not used retracted papers instead we have included three articles to elaborate the burden of listeriosis and its antibiotic resistance patern as suggested the reviewer. For your information, we have removed articles entitled “*Awofisayo A, Amar C, Ruggles R, Elson R, Adak G, Mook P, et al. Pregnancyassociated listeriosis in England and Wales. Epidemiology & Infection. 2015;143(2):249-56*” as we don’t believe the necesssity of citing articles for eligibility criteria.
