# Supplementary material for: A vigilant observation to pregnancy associated listeriosis in Africa: Systematic review and meta-analysis
Source: PLOS Glob Public Health. 2022 Oct 14;2(10):e0001023. doi: 10.1371/journal.pgph.0001023 (PMC10021409; doi:10.1371/journal.pgph.0001023)
Supplement: Attachment — Submitted filename: Response letter to editor of PGPH-2.docx [file pgph.0001023.s004.docx]

Editorial corrections

Dear Editors, we appreciated your efforts and gentle feedbacks.

In the previous revision, we have been replied point by points for comments given and hopefully the journal requirements were addressed. The recent email received is similar to the previous email received, 9 July 2022.
